# Impact of weather changes on hospital admissions for hypertension

**DOI:** 10.1038/s41598-022-09644-5

**Published:** 2022-04-05

**Authors:** Frederic Bauer, Janine Lindtke, Felix Seibert, Benjamin Rohn, Adrian Doevelaar, Nina Babel, Peter Schlattmann, Sebastian Bertram, Panagiota Zgoura, Timm H. Westhoff

**Affiliations:** 1 Department of Internal Medicine I, University Hospital Marien Hospital Herne, Ruhr-University of Bochum, Hölkeskampring 40, 44625 Herne, Germany; 2 Department of Medical Statistics, Informatics and Data Science, Jena University Hospital, Jena, Germany

**Keywords:** Cardiovascular diseases, Hypertension

## Abstract

Blood pressure (BP) shows a seasonal variation with higher levels at lower temperatures. Many hypertensives, however, report on BP disturbances rather in association with acutely changing weather conditions than with absolute temperatures. To date, the impact of changing meteorological parameters on hypertensive episodes remains elusive. We performed a retrospective time series regression analysis on 203,703 patients in three hospitals in Germany between 2010 and 2018, of whom 7362 patients were admitted for hypertensive disease. Numbers of daily admissions for hypertension were associated with metereological data obtained from three nearby weather stations. Data comprised temperature (mean, maximal, minimal and range within 24 h), athmospheric pressure, and precipitation. Changes of these parameters were calculated over a two and three day period. There was an inverse correlation between maximal daily temperature and the number of admissions for hypertensive disease, which remained significant both after adjustment for seasonality and week day in a spline model and in a constrained distributed lag model. A decrease of maximal temperature by 5 °C was associated with a 3% increase of risk for admission for hypertension and vice versa. There were no significant effects of precipitation and athmospheric pressure on the number of admissions. With regard to all observed metereological parameters, neither the change within two, nor within three days was consistently associated with the number of daily admissions. High temperatures are associated with lower numbers of hypertensive episodes requiring hospital admission. In contrast to the subjective perception of many hypertensive patients, however, acutely changing weather conditions are not associated with a higher risk of hypertensive emergency.

## Introduction

Many hypertensive patients report on blood pressure (BP) disturbances in times of changing weather conditions. So far, however, there is only data on the association of absolute temperatures and hypertension. Thus, BP shows a seasonal variation with lower levels at higher environmental temperatures and higher levels at lower temperatures^1–5^. Daytime BP is increased in winter, whereas there is an elevation of nighttime BP in summer^6^. The elevation of BP in wintertime is primarily attributable to low daytime temperatures. In contrast, the summertime elevation of nighttime BP is an indirect consequence to higher nighttime temperatures: It is considered to be related to poor sleep quality in warm summer nights^6^. The seasonal variation of BP is a global phenomenon affecting both sexes, all age groups, normotensive individuals, and hypertensive patients^1,4^.

Low and high temperatures are associated with increased cardiovascular mortality^7,8^. A recent study analyzed 74 million deaths in 13 countries and estimated that 7.7% of overall mortality was attributable to environmental temperature exposure^9^. High BP occurs more frequently in winter than in summer^10^. A likely physiological explanation is subcutaneous vasoconstriction leading to an increased total peripheral resistance and central blood volume.

Two studies have investigated the impact of ambient temperatures on hospitalization rates for hypertension^11,12^. Hospitalizations for hypertensive crises are less frequent during summer-time high temperatures. Low temperatures and thereby wintertime are associated with a higher number of hospitalizations. This phenomenon is of clinical relevance, since the decline of BP in summer may be associated with orthostatic symptoms and may thereby necessitate a reduction of antihypertensive medication. In contrast, an up-titration may be required in winter A consensus statement by the European Society of Hypertension Working Group on Blood Pressure Monitoring and Cardiovascular Variability summarized the current evidence on the seasonal variations of BP and provides recommendations for adjustment of antihypertensive treatment^1^.

Hence, a change of weather conditions induces compensatory vegetative reactions. It is conceivable, that these reactions are perceivable for some "meteorosensitive" subjects. Whereas the depicted evidence for seasonal long-term effects of temperature on BP is robust, the effects of short-term changes of metereological parameters on BP are unknown. Moreover, it remains elusive, whether such acute changes of temperature or athmospheric pressure are associated with an increased risk of hospitalization for hypertension. If so, there would be a clinical need to acutely adjust hypertensive treatment. Therefore, data on this topic are urgently required. The present work associates weather parameters from three meteorological stations in the Ruhr-region of Germany with the incidence of hospitalizations for hypertension in three large hospitals of this region. It examines for the first time, whether short-term changes of metereological parameters within three days predict an increased incidence of admissions for hypertension.

## Methods

### Protocol

Data on average daily ambient temperatures (T), precipitation (rainfall, hail or snowfall, R) and athmosperic pressure (P) for the observation period of January 1st 2010 to December 31st 2018 was obtained from the National Reference Institution (“Deutscher Wetterdienst”). Parameters were measured at three meteorological stations (Waltrop-Abdinghof, Gelsenkirchen-Buer, Essen-Bredeney). These meteorological stations were located closest to the participating hospitals with a distance of < 25 km each. Meteorological parameters comprised maximal, minimal and mean daily temperature (T_max_, T_min_ and T_mean_; °C), R (mm in 24 h) and mean P (hPa). Changes of these parameters from one day to the other were calculated as Δ_d2_T, Δ_d2_R and Δ_d2_P as well as changes within three days as Δ_d3_T, Δ_d3_R and Δ_d3_P. Moreover, the daily temperature amplitude T_range_ (T_max_ − T_min_) and the maximal temperature amplitude between two days T_range_max_ (T_max_ − T_min_ of two consecutive days: T_range_max_a_ = T_max_yesterday_ − T_min_today_, T_range_max_b_ = T_max_today_ − T_min_yesterday_) were calculated. Thus, there were eighteen meteorological datasets comprising data for each of the 3287 days in the observation period. The metereological data are available on request from the “Deutsche Wetterdienst” (www.dwd.de), the data on daily numbers of admissions for hypertension are available from the corresponding author on reasonable request.

We performed a systematic retrospective multi-center analysis using an electronic data extraction approach to identify subjects, who were admitted to hospital for hypertensive disease. Three hospitals in two cities (Herne, Witten) of the central Ruhr-region in Germany participated in the project. All hospitalizations for hypertensive diseases in these hospitals were extracted in a day-by-day manner using the diagnosis coded at discharge and submitted to the health insurance. Briefly, all subjects with a diagnosis related group (DRG) code "I10" or “I11” between 2010 and 2018 were identified. These DRGs comprise admissions for “hypertension” and “hypertensive emergency” and “hypertensive heart disease”. Thus, we included only patients, who attended the hospital for the primary diagnosis of hypertension. Admission for alternative diagnoses with hypertension as a comorbidity was an exlcusion criterion. Daily numbers of admissions in the three hospitals were added to an overall sum of hospitalizations. According to the local code of medical ethics, an ethics approval was not necessary (https://recht.nrw.de/lmi/owa/br_text_anzeigen?v_id=74120170406111640601). The local ethical committee of Ruhr-University Bochum decided that there was no need for ethical approval, since there was no personalized data (registration number 21-7424-BR). Data obtained from the three hospitals contained only numbers on daily admissions and numbers of diagnoses. Data did not obtain any additional personal information like age, gender, etc., either. Every hospital in Germany has to publish such data sets on numbers of patients and diagnoses on an annual basis as individual quality reports in order to make its competences and focuses transparently available. The study thereby followed the Declaration of Helsinki.

### Statistical analysis

In a first approach Spearman analysis was used to analyze the association between hospitalizations and weather parameters. Subsequently, a time series regression study was performed as proposed by Bashkaran et al.^13^ We made use of Poisson regression models with the number of admissions due to hypertension as dependent variable. In order to take the numerator i.e. the total number of hospital admissions into account we used its natural logarithm as an offset yielding a regression coeffcient equal to one. Thehe respective weather parameters were used as explanatory covariates in the regression model. Sometimes, the observed variability of the data is larger than the variability explained by a Poisson distribution, a phenomenon called “overdispersion”. To account for overdispersion we made use ofequasi Poisson models.

Since the study investigated metereological short term effects, data were adjusted for seasonality and long term trends by flexible spline functions. These are formed by polynomial—in this case cubic-curves, which are joined smoothly end to end to cover the full observation period. Thus, a set of basis variables was generated which are functions of the main time variable. These basis variables were included in the Poisson model. To generatie the spline basis, it is necessary to decide how many knots (join-points) there should be. The number of knots determines the number of end-to-end cubic curves. The more degrees of freedom, the more flexible the function is. Seven splines per year provide a a balance between adequate seasonality control without competing with the variable of interest to explain the short term variable of interest. We primarily used 62 knots [(Number of years × 7) − 1] and afterwards changed the number of knots as sensitivity analysis.

In order to take delayed effects into account we used a distributed lag model (sevens days) as part of a sensitivity analysis. In the distributed lag model (DLM) the series of the weather parameter is shifted foreward in time an re-fitted with the previous adjusted model. The lag time was increased from 0 to 7 days and the lag effects were adjusted for each other by entering the 0- to 7-day shifted series simultaneously in the model. Statistical significance was evaluated using partial Wald test with a significance level set to *p* < 0.05. These statistical calculations constitute a state of the art approach to investigate changes of metereological parameters on clinical endpoints and has been performed in analogous studies in the past^14,15^. Calculations were done with freely available software R^16^.

### Ethics declarations

The local ethical committee of Ruhr-University Bochum checked the project and decided that an ethical approval was dispensable (registration number 21-7424-BR).

## Results

### Patients

From January 2010 to December 2018, there were an overall number of 203,703 patients admitted to the internal medicine emergency rooms of the three hospitals. The mean number of daily admissions were 62 ± 26 per day with a minimum of 12 and a maximum of 137. 7362 patients were admitted for hypertensive disease between 2010 and 2018. The mean number of daily admissions for hypertensive disease was 2 ± 2 (range 0–14).

### Metereological data

**Figure 1:**
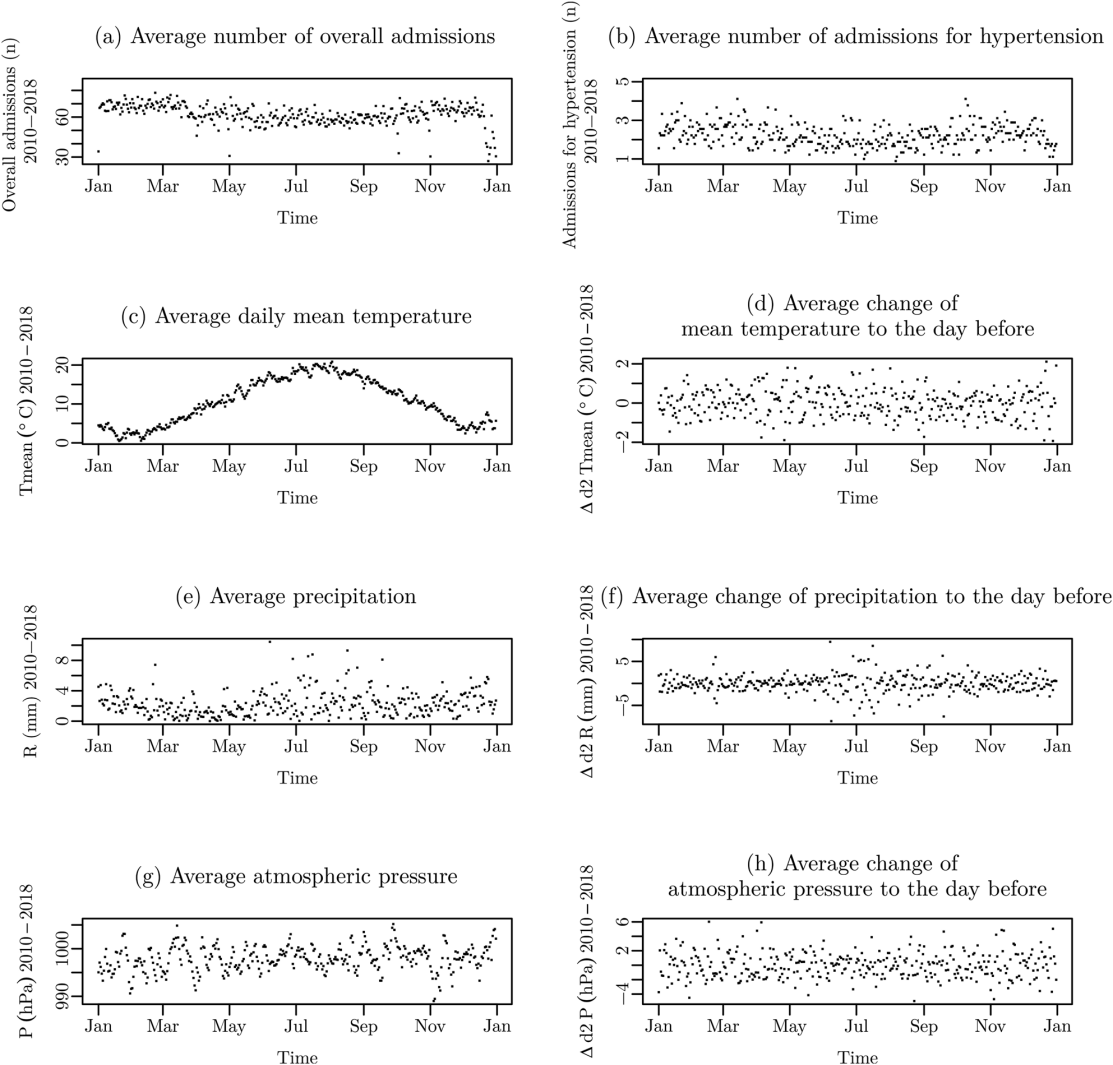
Mean annual course of (**a**) daily numbers of overall admissions, (**b**) daily numbers of admissions for hypertensive diseases, (**c**) daily mean temperature (T_mean_), (**d**) change of temperature to the day before (ΔT_mean_), (**e**) daily rainfall (R), (**f**) change of rainfall to the day before (ΔR), (**g**) daily mean athmospheric pressure (P), and (**h**) change fo athmospheric pressure to the day before (ΔP) between 2010–2018.

The mean temperature between 2010 and 2018 was 10.5 ± 6.8 °C. The maximal temperature was 36.7 °C (in July 2015 and August 2018). The mean daily temperature amplitude was 9.4 ± 4.7 °C, the maximal temperature amplitude between two days was 26.8 °C. The highest amplitudes occurred between March and April (> 21 °C). The mean daily precipitation was 2.3 ± 4.7 mm. Mean atmospheric pressure was 997.9 ± 8.7 hPa. From 2010 to 2018 the mean daily temperature increased from 8.9 to 11.5 °C. Except for 2018 (1.7 mm), mean daily precipitation was rather constant over the observation period (Ø 2.4 mm). Figure 1 presents the annual course of daily numbers of admissions, mean daily temperature, daily precipitation, daily mean athmospheric pressure and the corresponding changes of the meteorological parameters to the day before (Δ_d2_T, Δ_d2_R, Δ_d2_P).

### Association of metereological parameters and admissions for hypertension

**Figure 2:**
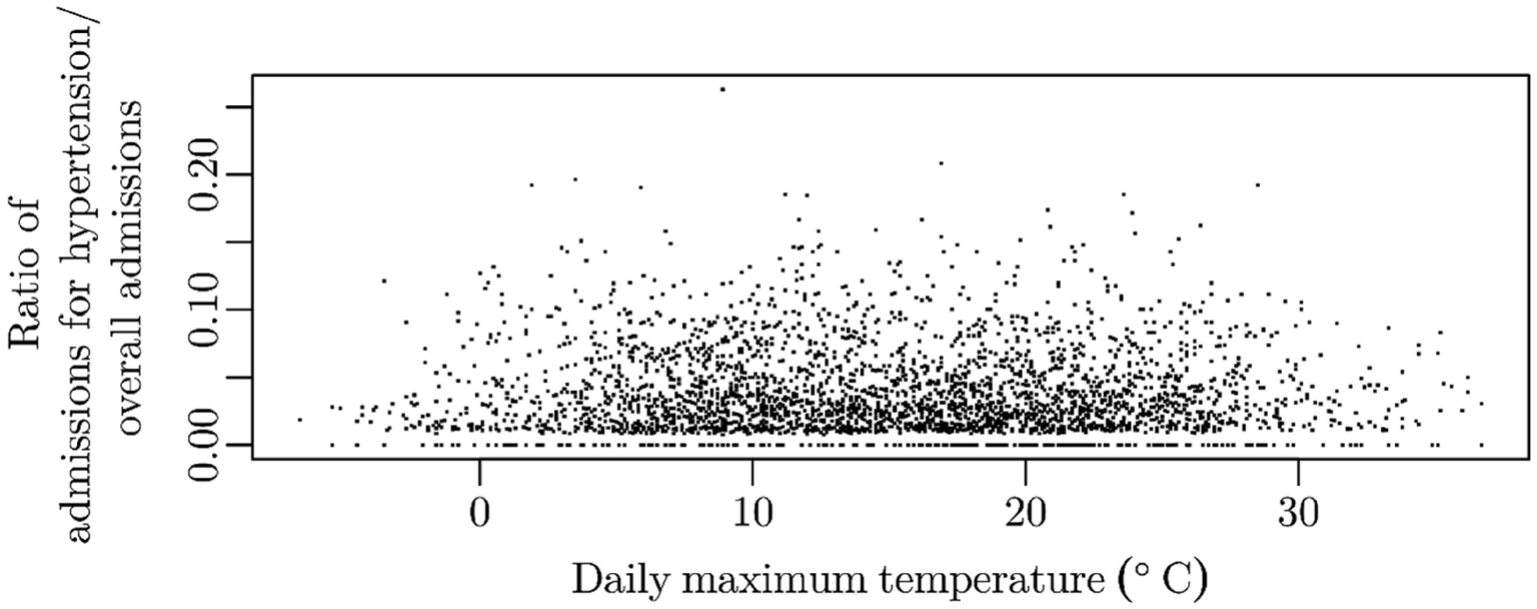
The association of daily maximum temperatures and the ratio of admissions for hypertension/ overall admissions.

Correlation analysis showed a significant correlation between T_mean_, T_max_, T_min_, T_range_, T_range_max_ and the number of admissions for hypertensive disease (Table 1). All Spearman correlation coefficients were negative, indicating that higher temperatures and temperature ranges are associated with a lower number of hypertensive emergencies. There was no correlation, however, for the changes of temperature from one day to the other, nor for changes within three days. Accordingly, neither precipitation, atmospheric pressure nor their changes from one day to the other or within three days were significantly associated with the number of daily admissions for hypertension (*p* > 0.05 each for R, P, Δ_d2_T, Δ_d2_R, Δ_d2_P and for Δ_d3_T, Δ_d3_R, Δ_d3_P). The association of daily maximum temperatures and the ratio of admissions for hypertension/ overall admissions is illustrated by Fig. 2.

**Table 1:** Correlation analysis (Spearman) of meteorological parameters obtained from three weather stations in the central Ruhr Region, Germany, between 2010 and 2018 and daily admissions for hypertensive disease.

Weather parameter	Mean values 2010–2018 (mean ± standard deviation)	Spearman coefficient of correlation (rho)	*p* value
T_mean_ (°C)	10.5 ± 6.8	− **0.059**	**0.001**
Δ_d2_T_mean_ (°C)	0 ± 2.3°	− 0.011	0.519
Δ_d3_T_mean_ (°C)	0 ± 3.3	0.010	0.585
T_max_ (°C)	**15.8 ± 8.0**	− **0.062**	**0.000**
Δ_d2_T_max_ (°C)	0 ± 3.1	− 0.014	0.416
Δ_d3_T_max_ (°C)	0 ± 4.2	0.008	0.651
T_min_ (°C)	**5.7 ± 6.1**	− **0.048**	**0.006**
Δ_d2_T_min_(°C)	0 ± 3.1	− 0.011	0.531
Δ_d3_T_min_ (°C)	0 ± 4.1	0.009	0.614
T_range_ (°C)	9.4 ± 4.7	− **0.040**	**0.023**
T_range_max_a_ (°C)	9.4 ± 4.6	− **0.039**	**0.026**
T_range_max_b_ (°C)	9.4 ± 5.1	− **0.041**	**0.020**
R (mm)	2.3 ± 4.7	0.019	0.265
Δ_d2_R (mm)	0 ± 5.9	0.009	0.611
Δ_d3_R (mm)	0 ± 6.3	0.002	0.925
P (hPa)	997.9 ± 8.7	0.010	0.551
Δ_d2_P (hPa)	0 ± 5.4	0.015	0.376
Δ_d3_P (hPa)	0 ± 8.1	0.011	0.513

*p* < 0.05 was regarded significant (bold type).

T—temperature, R—rainfall (precipitation), P—athmospheric pressure; Δ_d2_—change within two days, Δ_d3_—change within three days, T_range_: daily range of temperature, T_range_max_: maximum temperature range within two days (T_range_max_a_ = T_max_yesterday_ − T_min_today)_, T_range_max_b_ = T_max_today_ − T_min_yesterday_).

In a second step we analyzed the association of metereological parameters and daily admissions for hypertensive disease in an adjusted model accounting for seasonality and long-term effects (spline-model). After adjustment of the naive Poisson model for seasonality, long-term effects and weekday as confounders, only the association between T_max_ and hospital admissions for hypertension remained significant. The findings of this model are presented in Table 2.

**Table 2:** The association of metereological parameters with admissions for hypertensive disease in a quasi Poisson model adjusted for seasonality and long term trends with spline.

		RR	2.5% CI	97.5% CI	*p*
T_mean_	Per 5 °C incr	0.974	0.946	1.003	0.080
Δ_d2_T_mean_	Per 5 °C incr	0.978	0.937	1.022	0.328
Δ_d3_T_mean_	Per 5 °C incr	1.006	0.976	1.037	0.690
T_max_	**Per 5 °C incr**	**0.971**	**0.947**	**0.995**	**0.019**
Δ_d2_T_max_	Per 5 °C incr	0.986	0.955	1.018	0.395
Δ_d3_T_max_	Per 5 °C incr	1.002	0.979	1.027	0.851
T_min_	Per 5 °C decr	1.010	0.983	1.037	0.490
Δ_d2_T_min_	Per 5 °C incr	0.993	0.962	1.026	0.685
Δ_d3_T_min_	Per 5 °C incr	1.006	0.982	1.030	0.622
T_range_	Per 5 °C incr	0.976	0.950	1.002	0.066
T_range_max_a_	Per 5 °C incr	0.984	0.958	1.011	0.246
T_range_max_b_	Per 5 °C incr	0.978	0.955	1.001	0.057
R	Per 1 mm incr	1.001	0.997	1.005	0.646
Δ_d2_R	Per 1 mm incr	0.998	0.995	1.002	0.351
Δ_d3_R	Per 1 mm incr	0.998	0.995	1.001	0.188
P	Per 10 hPa incr	0.987	0.964	1.011	0.284
Δ_d2_P	Per 10 hPa incr	1.012	0.977	1.048	0.495
Δ_d3_P	Per 10 hPa incr	1.013	0.990	1.036	0.280

*p* < 0.05 was regarded significant (bold type).

T—temperature, R—rainfall (precipitation), P—athmospheric pressure; Δ_d2_—change within two days, Δ_d3_—change within three days, T_range_: daily range of temperature, T_range_max_: maximum temperature range within two days (T_range_max_a_ = T_max_yesterday_ − T_min_today)_, T_range_max_b_ = T_max_today_ − T_min_yesterday_), inc—increase, decr—decrease, *p*—*p* value, RR—relative risk, CI—confidence interval.

There was a significant inverse association of T_max_ and the number of daily admissions for hypertension (relative risk, RR, per 5 °C increase 0.971, 95% CI 0.947–0.995, *p* = 0.019). Thus, the risk for admission to hospital for hypertension decreases by 3% per 5 °C increase of T_max_. Vice versa, the risk for admission is increased by 3% with a 5 °C decrease of T_max_.

There were analogous trends for T_mean_ and T_range_ (RR per 5 °C increase 0.974, 95% CI 0.946–1.003, *p* = 0.08 and RR 0.976, 95% CI 0.950–1.002, *p* = 0.066 respectively), which did not reach, however, statistical significance. A decrease of T_min_ was not statistically significantly associated with an increasing risk of hospital admissions for hypertension (RR per 5° C decrease 1.010, 95% CI 0.983–1.037, *p* = 0.490).

Neither atmospheric pressure, nor precipitation had a significant effect. There was no effect of interday changes of any of these parameters either. These results did not change significantly after adding weather parameters as confounders in the models: Thus, e.g. the findings for temperature effects remained significant after controlling for athmospheric pressure and precipitation.

Potentially delayed effects were calculated with constrained distributed lag models (DLM) as part of the sensitivity analysis. The protective effect of increasing temperature was reproducible in the DLM. Per 5 °C increase of T_mean_ the RR_cumulative_ is 0.602 with an 95% CI of 0.387–0.938, for T_max_ the RR_cumulative_ was 0.671 per 5 °C increase with an 95% CI of 0.462–0.973. This means that the cumulative effect of increasing temperature after a 7 day lag period is a risk decrease up to 40% per 5 °C increase.

The sensitivity analysis also included changing the amount of control for seasonality and long term trends by changing the number of knots in the spline based approach as proposed by Bashkaran et al.^13^ After reducing the number of knots from 62 to 40 as well as after increasing the number from 62 to 75 the protective effect of T_max_ in the adjusted model remained. However for T_mean_ and T_max_ the effect in the lag model was no longer significant.

## Discussion

Many patients with hypertension report that they perceive short-term weather changes by increasing BP. This subjective view, however, has never been investigated in an adequate scientific manner. Whereas there is robust evidence for long-term, seasonal effect of changing temperatures on BP, these data are lacking for short-term changes of weather conditions^1,3–6^. The present study constitutes the first systematic analysis of the association of short-term weather changes and hospitalizations for hypertension. Our findings show that neither a change of temperature, athmospheric pressure, nor rainfall within two or three days is associated with the incidence of hospital admissions for hypertension. The analysis comprises a period of nine years and more than 200,000 patients in the emergency rooms of three large hospitals. The observed nine years cover both years with outstandingly warm summers (e.g. 2015 and 2018) and cold winters (e.g. 2010–2012). Thus, the findings may be regarded as rather representative for a middle European region.

The study did not examine BP itself. Instead, the daily incidence of hospital admissions for hypertension was analyzed as a surrogate parameter of severe hypertensive episodes. In fact, some of the hospitalizations may have taken place primarily for diagnostic reasons, e.g. an exclusion of secondary causes of hypertension. Since these diagnostics usually take place in an outpatient setting, however, it may be assumed that the majority of cases indeed represent hypertensive emergencies. Moreover, admissions for diagnostic reasons are unlikely to be affected by weather conditions. Hence, the study does not exclude a potentially mild effect of changing meteorological parameters on BP. It does exclude, however, that short-term changes are associated with substantial increases of BP necessitating hospitalization.

Our study supports the clinical relevance of previous findings on the association of absolute temperatures and BP^1,2,11,17–24^. In 1961 there was a highly ranked study showing that BP in men is lower in summer than in wintertime^3^. This finding was confirmed e. g. in the PAMELA cohort, a population-based study in Ontario, Canada, and elderly individuals in the Three-City Study^4,8,11^. This finding is supposed to be primarily mediated by peripheral vasoconstriction in colder temperatures. Our data confirm that the long-term changes of metereological parameters translate into a higher hospitalization rate for hypertension in wintertime.

This central finding was consistent in both mere correlation analysis and all applied adjusted models accounting for seasonality, week day, athmosperic pressure, precipitation as well as in the lag model. In order to assess the quantity of this effect, we analyzed the assocation of a 5 °C decrease of maximal temperature on hospital admission. This decrease was associated with a 3% increase of risk for admission, whereas a 5 °C increase lowered the risk by 3%. The correlation between mean and minimum daily temperatures with the number of hospital admissions lost statistical significance in the adjusted models. The maximal daily temperature is usually reached during daytime, whereas minimal temperatures occur at nighttime. Nighttime temperatures, however, are likely to have a lower effect on BP, since they affect the sleep period with people being inside. As described above, there is a summertime elevation of nighttime BP, which is considered to be related to poor sleep quality in warm summer nights^6^.

Atmospheric pressure is the most objective weather parameter because it is independent of outdoor or indoor activities. The association of atmospheric pressure and BP has been investigated before with conflicting findings. Thus, a significant inverse relationship between atmospheric pressure and systolic BP has been described for hypertensive patients in individual reports^25^. Our study and the majority of previous investigations found no correlation of atmospheric pressure with BP changes^1,2^.

To date, there are only three studies that have investigated the impact of temperature on hypertension-related hospitalizations. A recent Serbian study found no association between three weather parameters (temperature, atmospheric pressure, and relative humidity) and hospitalizations for hypertension^26^. The investigation covered a period of two years in the city of Novi Sad. The overall number of admissions for hypertension was 264 and thereby substantially lower than in the present investigation (n = 7362). In contrast, a study in Ontario, Canada, found that cold temperatures (1st percentile compared to the temperature with minimum risk of morbidity) were associated with a 37% increase in hypertension-related hospitalizations whereas no significant association with hot temperatures was observed^12^. Accordingly, an investigation in New York reported that summer-time high temperatures were associated with reduced risk of hypertension-related hospital admissions^27^. Unfortunately, it did not evaluate any possible effects from cold temperatures. None of these studies, however, evaluated the effects of short-term metereological changes on BP or hospital admissions.

Our study investigated the effects of short-term changes of metereological parameters on hospital admissions for hypertension for the first time and exruled a significant association. On the one hand, this might be intriguing, since the subjective perception of changing weather is frequently stressful. This stressful perception could indeed have led to an increase in sympathetic tone. On the other hand, it may be questioned, in how far subtile changes in temperature or athmospheric pressure may elicit a clinically relevant effect on the autonomic nervous system. Our data disprove that this psychological phenomenon has a substantial impact on vasomotor tone leading to an increased risk of hospitalization for hypertension. Moreover, many patients decide to attend a hospital only in case of long-lasting complaints. Hence, resolution of a potential short-term increase on BP might have prevented the need for hospitalization. Even if so, however, the present large-scale data exclude clinically relevant hypertensive adverse events in context of short-term weather changes.

Our study is limited by the lack of data on BP itself. Data on BP cannot be derived from a DRG based case extraction. This approach, however, allows to investigate a very high number of subjects. Moreover, it allows to analyze the clinical relevance of potentially changing BP in terms of a "harder" cardiovascular endpoint, namely the need for hospitalization for substantially increased BP values.

The present analysis constitutes the first investigation on the association between acute changes of weather parameters and hypertension-associated hospitalizations. It shows that short-term changes in weather do not increase the risk for hypertensive emergencies. It supports, however, the seasonal long-term association of low temperatures with a higher risk of hospitalization and vice versa.

## Conclusion

The findings of our investigation may be of practical clinical relevance. On the one hand, physicians should inform hypertensive patients about a potential need of an intensified medication in colder seasons and should encourage them to check their BP eventually. On the other hand, they can inform them that acute changes in weather conditions will have little effect on their BP.

## Data Availability

The metereological data are available on request from the “Deutsche Wetterdienst” (www.dwd.de), the data on daily numbers of admissions for hypertension are available from the corresponding author on reasonable request.
